# Promoting health with human rights in Indonesia

**DOI:** 10.2471/BLT.16.031216

**Published:** 2016-12-01

## Abstract

Nafsiah Mboi tells Fiona Fleck why technical know-how, insistence on human rights and gentle persuasion were essential ingredients for the modernization of public health in Indonesia.

Q: Why did you study medicine?

A: When I was about seven, the first doctor came to live in our village and we became neighbours. I was impressed and inspired when I saw how he treated patients and they went home relieved. At that time it was almost unthinkable for a woman to apply for medical school, but I did so with the encouragement of my family. In 1964, when I graduated, I became the first woman doctor from the Buginese Makassar ethnic community. After graduation I was stationed in a remote and poor area in East Nusa Tenggara province. European missionary doctors had been posted there from time to time, but I was the first Indonesian woman doctor to work there. In 1967, I applied to do obstetrics–gynaecology. At that time, there was gender discrimination in medicine so both obstetrics–gynaecology and surgery were closed to women. I always loved children so I switched to paediatrics. Today women in Indonesia can specialize in any field they like.

Q: What drew you to public health?

A: I fell in love with public health while my husband was the governor of East Nusa Tenggara (1978–1988). It was one of the poorest provinces in Indonesia and had high rates of infant and maternal mortality, malnutrition and infectious diseases. Local health budgets were tiny, and the health infrastructure was virtually non-existent. As governor’s wife, I was responsible for the village family welfare movement and community groups. We focused on three areas: health, education and income generation. During my 10 years there was some progress, including a reduction in the diseases of poverty. For example, deaths from diarrhoeal diseases declined as people learned the importance of hygiene, breastfeeding and how to treat dehydration in children. I saw how much people can do to improve health but also how much more needed to be done.

Q: How did you become involved in the fight against HIV?

A: When I did my master’s degree in public health, most of my fellow students were from Africa and their countries were struggling with rising numbers of people with HIV (human immunodeficiency virus) infection. Then I spent a year at Harvard School of Public Health researching the diseases of the urban poor, including HIV. The late Jonathan Mann, who had led the Global Programme on AIDS at the World Health Organization (WHO), was an important friend and mentor at Harvard. He taught me about human rights violations in health, particularly the detention of people with HIV. He told me again and again: “When you go home, you must do something about HIV”.

Q: How did you do this?

A: When I returned to Indonesia in 1991, very few people had heard about AIDS (acquired immune deficiency syndrome) and almost no one was interested. A few physicians and I started to raise awareness about HIV infection among medical colleagues in government as well as civil society and the media. Our message was: “Wake up, this is coming to us and even though you think we are good religious people we have to start talking about AIDS openly”. In 1992, I became a member of parliament and continued with AIDS advocacy. After a study tour in Thailand, we developed a draft national strategy on HIV. After nine months of consultation with government ministries and 13 revisions, the strategy was approved in 1994. The National AIDS Commission was established, but still not much happened. We knew that people were injecting drugs, which was illegal, and there was a lot of unprotected sex. HIV was a very real threat in Indonesia but it was not until 2003 that we managed to get the government to listen to us.

“When I returned to Indonesia in 1991, very few people had heard about AIDS and almost no one was interested.”

Q: How did you succeed in doing that?

A: The Chair of the National AIDS Commission called a meeting to brief members about AIDS, particularly the commission’s vice chairs, who were also the national ministers of health, social affairs, education, religion and home affairs, and the head of the National Family Planning Board and the chair of the Parliamentary Committee on Health. Most of them knew little about AIDS and had shown little interest. We gathered in January 2004 in the village of Sentani in the province of Papua, which had the country’s highest HIV prevalence. The meeting ended with a declaration, the Sentani Commitment. It was the first joint government and civil society call for recognition of the urgent need to respond to HIV and for commitment to promote condom use, harm reduction for injecting drug users, access to antiretroviral treatment and efforts to reduce the stigma and discrimination associated with HIV.

Q: You became head of the National AIDS Commission in 2006. How did you step up the fight against AIDS?

A: The Sentani Commitment didn’t solve all of our problems but gave us a basis for working more assertively with government while continuing outreach and collaboration with civil society and the people most at risk of HIV infection. The effort was hampered by widespread ignorance and discrimination against people living with HIV. Nonetheless, we continued to work closely with the key populations affected by HIV, particularly sex workers, injecting drug users and men who have sex with men. We also worked with government agencies to increase the availability, acceptability and utilization of services for HIV prevention and treatment.

Q: How did you change attitudes?

A: For example, as secretary of the National AIDS Commission I invited officials from the police and the National Narcotics Board to a meeting on injecting drugs. We recognized that this was illegal but the fact was there were about 230 000 people who injected heroin. I said: “They are our children and we, as government, have promised to protect them and promote their human rights and health.” The drug enforcement officials said: “We can’t work with these people, they are criminals.” But I replied: “We can’t just let them die and they will die, if we do not act.” After a pause, one policeman spoke up and said: “You are right, my son is a drug user and I don’t know what to do. Can we save him?” I explained how harm reduction worked: through the provision of clean needles and condoms. Eventually we adopted this approach thus reducing the spread of HIV and saving the lives of many injecting drug users in Indonesia.

Q: What was your involvement in a law calling for every baby to be breastfed or given donor breast milk exclusively for the first six months of life?

A: When I returned to Indonesia in 2003 after working at WHO, formula milk was being promoted aggressively. Even midwives and paediatricians were recommending it. According to government data, Indonesia has always had high rates of breastfeeding, but these were declining rapidly. The 2009 law on the promotion of breastfeeding was a good policy, but it was not fully implemented. So when I became health minister I took various actions. I worked directly with professional organizations of midwives, obstetricians and gynaecologists, and paediatricians and reminded them of their responsibility for the well-being of mothers and their babies, and asked them to promote breastfeeding. I called milk formula distributors and told them they could no longer use the phrase “breast milk substitute” because nothing can substitute breast milk. We also banned all forms of marketing and promotion of formula for babies under six months of age.

“I called milk formula distributors and told them they could no longer use the phrase ‘breast milk substitute’ because nothing can substitute breast milk.”

Q: What was your contribution to rolling out universal health coverage?

A: Indonesia passed a law in 2004 that led to the establishment in 2014 of a national social health insurance scheme. Before 2004, only civil servants, police officers and military personnel were covered by a public social insurance scheme. This scheme was expanded gradually to cover the poor and include maternity care. As Minister of Health, I was fortunate to be directly involved in the preparations throughout the final year before the launch of the comprehensive system and the first year of operations. The challenges were many. Indonesia is a huge country with 250 million people, spread across 17 000 islands. Responsibility and budgets for health are decentralized and health coverage is uneven. We worked closely with local government in all provinces with health-care professionals to move toward our shared goal of equitable and quality health care for all Indonesians.

Q: What steps did your country take to curb the influence of the tobacco industry and what was your role in this?

A: Despite the efforts of many people including myself as health minister, Indonesia has still not ratified the WHO Framework Convention on Tobacco Control (WHO FCTC). We have, however, made progress in terms of taking some measures to control tobacco use. As health minister, I used the right to health to justify increasing public education on the harm caused by smoking. After my term as health minister, I joined the National Commission on Tobacco Control and together with local government, civil society and the private sector we strengthened advocacy and raised awareness. The battle is not over yet, but we have made progress. More people now support tobacco control including influential people in the media. In Jakarta, tobacco advertising on billboards is banned and smoking is restricted in public places.

Q: Since then, you have joined the regional fight against malaria. What are the challenges?

A: I became the Leaders’ Envoy of the Asia Pacific Leaders’ Malaria Alliance (APLMA) last year. The APLMA was established by the East Asia Summit (EAS) in 2013. In 2014 the APLMA pledged to eliminate malaria in the region by 2030. Working with malaria programmes in countries and international partners, including WHO Regional Offices, and the Global Malaria Programme, we have developed a roadmap to achieve that goal. Our two main challenges are the last remaining pockets and the emergence of resistance to artemisinin, the treatment of choice. If not contained, a resurgence of malaria will occur not only in our region but elsewhere. That’s why there’s a growing consensus that malaria elimination and, ultimately, eradication must be our goals. Thanks to its political commitment to sustained policy, programmes and budget to finish the job, Sri Lanka has achieved this. I am sure other countries will follow suit. 

